# Home Energy Management System Incorporating Heat Pump Using Real Measured Data †

**DOI:** 10.3390/s19132937

**Published:** 2019-07-03

**Authors:** Zhengnan Cao, Fergal O’Rourke, William Lyons, Xiaoqing Han

**Affiliations:** 1 Centre for Renewables & Energy, School of Engineering, Dundalk Institute of Technology, A91 K584 Dundalk, Co. Louth, Ireland; 2 School of Engineering, Dundalk Institute of Technology, A91 K584 Dundalk, Co. Louth, Ireland; 3 College of Electrical and Power Engineering, Taiyuan University of Technology, Taiyuan 030024, China

**Keywords:** home energy management system (HEMS), heat pump (HP), particle swarm optimisation (PSO), indoor thermal model, demand side management (DSM)

## Abstract

The demand for electricity has been rising significantly over the past years and it is expected to rise further in the coming years due to economic and societal development. Smart grid technology is being developed in order to meet the rising electricity requirement. In order for the smart grid to perform its full functions, the Energy Management Systems (EMSs), especially Home Energy Management Systems (HEMS) are essential. It is necessary to understand the energy demand of the loads and the energy supply either from the national grid or from renewable energy technologies. To facilitate the Demand Side Management (DSM), Heat Pumps (HP) and air conditioning systems are often utilised for heating and cooling in residential houses due to their high-efficiency power output and low CO_2_ emissions. This paper presents a program for a HEMS using a Particle Swarm Optimisation (PSO) algorithm. A HP is used as the load and the aim of the optimisation program is to minimise the operational cost, i.e., the cost of electricity, while maintaining end-user comfort levels. This paper also details an indoor thermal model for temperature update in the heat pump control program. Real measured data from the UK Government’s Renewable Heat Premium Payment (RHPP) scheme was utilised to generate characteristic curves and equations that can represent the data. This paper compares different PSO variants with standard PSO and the unscheduled case calculated from the data for five winter days in 2019. Among all chosen algorithms, the Crossover Subswarm PSO (CSPSO) achieved an average saving of 25.61% compared with the cost calculated from the measured data with a short search time of 1576 ms for each subswarm. It is clear from this work that there is significant scope to reduce the cost of operating a HP while maintaining end user comfort levels.

## 1. Introduction

According to SmartGrid.gov, the U.S. Department of Energy (DOE) information gateway, the efficiency, security and reliability are increasing with the development of communication, control, and automation technologies [1]. With the ever-changing pace of industrialisation processes, and entering the era of big data, cloud computing and mobile internet, new energy challenges have arisen. The smart grid technology is being developed to address these challenges and there has been an increasing number of publications in this critical area with the industry showing great interest in recent developments [2].

In order for the smart grid to achieve appropriate functionality, Energy Management Systems (EMSs) are required to play a major role [1]. EMSs basically attempt to gather information about the indoor environment, human behaviour, and load conditions, and apply suitable control to manage the electrical loads in order to maximise the efficiency of electricity use and reduce costs for the end user. The EMSs in the context of the smart grid, have developed a great number of new functions compared with a traditional EMS. An EMS can be divided into two major categories, one is for small-scale buildings and organisational EMS (single unit EMS), the second type is a large-scale EMS (whole-system EMS), like micro-grid EMS or EMS used by the utility [3]. This work focuses on a Home Energy Management System (HEMS) which belongs to the single unit EMS category. In a HEMS, in order to optimally schedule the residential loads to achieve minimum electricity cost, three approaches are often used: mathematical optimisation, meta-heuristic searches, and heuristic methods. The Particle Swarm Optimisation (PSO) algorithm is a popular meta-heuristic method that is efficient and easy to implement. In the smart grid context, Heat Pumps (HPs) are regarded as a critical load of Demand Side Management (DSM). The flexibility of smart grid operation can be achieved by utilisation of HPs along with thermal storage using the thermal inertia of houses. HPs also facilitate the utilisation of renewable energy under variable electricity prices [4].

There is a body of research in the published literature that utilised PSO-based methods in HEMS. Some papers utilised the standard PSO algorithm. Liao and Yang developed a novel Electron Drifting Algorithm (EDA) for a Battery Energy Storage System (BESS) operation management in HEMS [5]. The proposed EDA is compared with PSO, Differential Evolution (DE), and Artificial Bee Colony (ABC). According to the convergence curves obtained, the PSO converges faster than the other three algorithms. However, it has a higher probability to fall in a local optimal solution when compared with the other three algorithms. Mangiatordi et al. investigated the use of PSO in load scheduling and Peak to Average Ratio (PAR) reduction in HEMS [6]. The PAR is defined as the ratio of the daily peak energy consumed to the daily average energy consumed. It is indicated by the simulation results that the probability of finding the optimal solution increases by using a number of swarms. The proposed algorithm has a fast convergence speed that is able to find the optimal solution in a few hundreds of milliseconds. Gudi et al. proposed a dynamic Distributed Energy Resources (DER) management system using PSO in a simulation tool based on DSM to minimise electricity cost [7]. The efficiency of energy utilisation has been increased by 16.4% by using the proposed PSO approach.

Binary Particle Swarm Optimisation (BPSO) is a popular variant of PSO that is adopted by a number of applications due to its ease of implementation. Shah et al. proposed a HEMS using Multi-agent System (MAS) under a Time of Use (TOU) pricing tariff [8]. Priority techniques are used together with the electrical supply system. The BPSO algorithm is used for load scheduling in the proposed system. Two priority techniques are used to calculate the demand of the end users including Priority of User Comfort (PUC) and Priority of Lowest Consumption (PLC). In BPSO, the particles exist in the form of a string of binary numbers. Each particle (bit in the string) is required to make a binary decision, either one or zero. It is indicated by the simulation results that BPSO performs better with PUC in terms of electricity cost and PAR reduction compared to PLC. Optimal energy efficiency is achieved by consuming a minimum amount of energy at both high and low peak hours. Reducing the PAR by reducing peak hour demand is a critical method to realise optimal energy efficiency [9]. Rahim et al. compared and assessed the performance of a number of HEM controllers under Real-Time Pricing (RTP) [10]. The algorithms compared include Genetic Algorithm (GA), Bacterial Foraging Optimisation Algorithm (BFOA), BPSO and Ant Colony Optimisation Algorithm (ACO). The average cost for GA, BFOA, BPSO and ACO are 95.58%, 81%, 90.4% and 76.48%, respectively, with respect to the unscheduled electricity cost. The PAR obtained under ACO and GA are relatively higher than BFOA and BPSO which indicate the trade-off between cost and PAR. Zhou et al. proposed a real-time optimal appliances scheduling approach using BPSO with the participation of both energy suppliers and end users and the inclusion of renewable energy sources [11]. Javaid et al. proposed a HEMS controller based on four heuristic algorithms: BFOA, GA, BPSO, and Wind Driven Optimisation (WDO) [12]. In addition, a hybrid algorithm called Genetic Binary Particle Swarm Optimisation (GBPSO) is proposed by combining GA and BPSO. The proposed algorithms are tested in RTP environment including necessary home appliances. It can be seen that BPSO based HEMS controller achieves 36% cost reduction which is relatively better compared with other algorithms. The proposed GBPSO based HEMS controller outperforms all other algorithms in terms of both cost and PAR reduction. For the single home scenario, GBPSO achieves the highest cost reduction of 37.76% while the cost reduction of BFOA, GA, BPSO, and WDO are 6.99%, 4.2%, 36.87%, and 32.29%, respectively. BPSO and GBPSO achieved similar cost reduction. However, GBPSO significantly curtails PAR by 43.08%.

Research has also been carried out that utilised both the standard PSO and the BPSO algorithm. Pedrasa, Spooner and MacGill proposed an energy service simulation platform using hybrid PSO aiming to maximise the benefits of energy services and minimise electricity cost by the optimal scheduling of DERs in a smart home [13]. The details of the hybrid PSO is as follows. Battery charging/discharging, heating power and starting times of the pool pumping periods are scheduled using real-valued standard PSO. The state of the pool pump (running or not) is set by the BPSO according to the calculated starting time. A smart home case study is conducted. It can be seen by the results that the proposed algorithm can perform effective management under TOU and RTP Tariffs. The proposed algorithm is also capable of DER management aiming to reduce electricity cost and maximise electricity export when the net feed-in tariff is available. Gudi, Wang and Devabhaktuni utilised BPSO for load scheduling in HEMS [14]. Renewable energy sources are included and the distribution of energy obtained from these sources is optimised using PSO. It is indicated by the results that the utilised BPSO for load scheduling achieved a cost saving of 33%, and the optimised resource management using PSO reduced the cost by 16–17%.

A number of papers incorporated HP in their developed HEMS. De Angelis et al. proposed an algorithm based on the mixed integer linear programming to minimise electricity consumption of different tasks and manage the renewable energy sources. [15]. The algorithm optimally schedules the loads while keeping the comfort level for the end users. A thermal model based on heat-pump usage has been included in the system. Simulations have been conducted using real data. The results indicate the effectiveness of the algorithm in terms of cost savings. Anvari-moghaddam, Monsef and Rahimi-kian developed a multi-objective mixed integer nonlinear programming model to optimise energy usage in a smart home, while considering end user comfort [16]. Simulations were conducted using real data for different scenarios and comparison were conducted between the obtained results. Asare-Bediako, Kling and Ribeiro proposed a HEMS based on multi-agent based architecture [17]. The authors proposed four strategies for optimization–comfort, cost, green (energy-efficient) and smart (demand side management). The aim of the strategies is to save energy and reduce electricity cost while providing flexible control to the end users and additionally facilitate the management of the electricity network by the utility companies. Simulations were conducted using MATLAB and JAVA/JADE platforms. Heat pump was included as one of the load. The results showed that energy consumption of the loads can be optimised within comfort range defined by the end users. Asare-Bediako, Ribeiro and Kling conducted research on HEMS using MATLAB/Simulink model for a typical Dutch household [18]. The type of feed-in tariff had a strong influence on the cost optimisation. The results show effectiveness for peak shaving and minimisation of network losses when connecting a number of households. Zhao et al. developed a HEMS with different load profiles incorporating PV system, energy storage and DC loads [19]. The proposed HEMS aims to optimise the usage of local renewable energy and minimise the waste of energy during AC-DC conversions and battery charging and discharging. The performance of the HEMS was evaluated on different households with and without electric heating. The electric heating includes both electric resistance heating and HPs. Investigation was conducted about battery behaviour, characteristics of AC and DC conversion. It is indicated by the results that load profiles have a large influence on electricity cost reduction and the most cost reduction can be achieved by end users with electric heating load and suitable size energy storage. The daily electricity cost reduction for the overnight heating, daytime heating, and evening heating are all around £0.33 compared with the unscheduled case without HEMS, which is 4.86%, 4.43%, and 4.15% reduction, respectively. Yoon, Baldick and Novoselac developed a Dynamic Demand Response Controller (DDRC) to change the temperature setting of a HP according to electricity retail price and reduce the load at the peak times [20]. Furthermore, a detailed single family house model was developed using OpenStudio and EnergyPlus. The proposed DDRC was built in MATLAB/SIMULINK and connected to the EnergyPlus model. A hypothetical real-time retail price based on the wholesale price in the ERCOT market in Texas in 2011 was utilised. The results indicate that the peak loads and electricity cost can be reduced while keeping thermal comfort. Setlhaolo, Sichilalu and Zhang utilised an energy hub framework to model the interaction among electricity and natural gas incorporating HP water heater, different energy sources and considering CO_2_ emission reduction [21]. There are two parts of this work. In the first part, the operation of the Combined Heat and Power CHP, Photovoltaic and storage system were optimised under changing electricity price. The second part investigated how to perform optimal load management in the proposed residential energy hub model. In order to achieve this, the modelling of a HP water heater was considered. The results also indicated that CO_2_ signal is able to encourage end users to shift or reduce loads during peak hours and reduce the electricity cost and carbon emissions.

Among all literature reviewed that incorporated a HP, no paper has yet utilised a particle swarm optimisation algorithm to optimise the operation of the HP in a HEMS. The optimisation algorithms utilised in the literature include the mixed integer linear programming, mixed integer nonlinear programming, multi-agent based architecture, or simple thermostat set-point control. The mixed integer linear programming and mixed integer nonlinear programming are both mathematical optimisation, they were utilised by De Angelis et al., and Anvari-Moghaddam et al. [15,16,19]. The computational costs of these mathematical optimisation approaches are often quite high and it is also quite complex to solve these mathematical problems [22]. Multi-agent based architecture is a heuristic method, Asare-Bediako, Kling and Ribeiro built their multi-agent based architecture simulation model using MATLAB and JAVA/JADE [17]. The model is complicated and requires a great amount of work to build. Yoon, Baldick and Novoselac adopted simple set-point temperature control which can only change the thermostat set-point by 1 degree Celsius according to a preset electricity price threshold [20]. This approach is too simple and one preset threshold cannot fully utilise the hourly changing TOU electricity price to achieve significant electricity cost savings. Due to the drawbacks of the above-mentioned optimisation algorithms, this current research adopted PSO algorithm which is efficient and only requires a few lines of code to implement. This current work is based on the conference paper titled ‘Home energy management system incorporating heat pump’ presented at the 2018 12th International Conference on Sensing Technology (ICST) [23]. This current work presents a purposely-developed program for a HEMS using the standard PSO algorithm with a HP as the load. This paper also utilises three variants of PSO which are compared in another U.S. HEMS case study conducted by the authors [24]. The three variants are Crossover Subswarm PSO (CSPSO), Quantum Particle Swarm Optimisation (QPSO) and Quantum Particle Swarm Optimisation Procedure with Lévy flights (QPSOL). CSPSO was proposed by the authors in the U.S. case study, QPSO and QPSOL were improved by the authors in the U.S. case study. This paper aims to compare the performance of standard PSO algorithm and its three variants to show the improvement of the variants compared with the standard PSO and prove that CSPSO has the overall best performance in terms of electricity cost reduction and search speed. The derivation of the indoor thermal model is presented in order to update the indoor temperature every hour under the influence of the HP. This paper utilises HP related data from the UK Government’s Renewable Heat Premium Payment (RHPP) scheme to generate characteristic curves of Coefficient of Performance (COP) versus outdoor temperature and heat pump hourly heat output versus outdoor temperature. These characteristic curves are then used to generate data for five winter days in 2019. These performance curves are utilised in order to conduct optimisation of HP operation.

This paper is structured as follows: in Section 2, the HP is briefly introduced and an overview of PSO and its variants are given, the derivation of the indoor thermal model is then presented. The data utilised and the program developed are described in detail. In Section 3, the results of the chosen algorithms are presented and compared. A discussion involving comparisons with works in the literature is presented in Section 4. Finally, the key conclusions are given in Section 5.

## 2. Materials and Methods

In this section, the materials and methods used to conduct this proposed research are discussed.

### 2.1. Heat Pump Overview

HP technology is becoming increasingly popular for heating and cooling in residential houses due to its high efficiency and low CO_2_ emissions [4]. The CO_2_ emissions when the HP is operating depend on the COP of the HP and the CO_2_ emissions of electricity generation type from the utility company, i.e., where more renewable energy technologies are used for electricity generation, there are less CO_2_ emissions. The CO_2_ emissions of HPs are lower compared to other heating technology systems due to its high COP and can be further reduced under high renewable energy mix. A long term goal was set in the Paris Climate Change Conference 2015 for reduction of coal-fired power plants [25], which will decrease the CO_2_ emissions for electricity generation and in turn reduce the CO_2_ emissions during HP operation. Approximately 800,000 electrically driven HPs have been sold in the EU per year from 2010 to 2015 resulting in a total number of 7.5 million installed HPs [26]. By utilising a vapour compression cycle, the heat is taken from low-temperature sources like air, ground, lakes or sea water and released in residential houses by the HP at a higher temperature for space heating and/or hot water usage. It is indicated by Chua et al. [27] that with the development of HP technology, has resulted in an increase in the COP. Additionally, it is indicated by the simulated results of a German renewable energy system that the HP significantly reduced the CO_2_ emissions compared to other energy sources [28,29]. Cockroft and Kelly investigated the potential of CO_2_ emission reduction of air source HPs in a simulation for the UK in a 2050 scenario [30]. They concluded that comparing with stirling engine micro-Combined Heat and Power devices (CHP), Internal Combustion Engine (ICE) micro-CHP devices and fuel cells, the potential of CO_2_ emission reduction of air source HPs is the greatest compared to using a condensing boiler and grid electricity.

### 2.2. Particle Swarm Optimisation Overview

PSO was first proposed by social psychologist Kennedy and electrical engineer Eberhart [31]. The algorithm was inspired by the modelling and simulation results of bird flock behaviour. The mathematics of the PSO algorithm is quite simple so that the computation cost is relatively low with low requirements for memory and processor speed. At the same time, the effectiveness of finding the optimum solution is at the same level compared with similar meta-heuristic algorithms, for example, the Genetic Algorithm. A book called Swarm Intelligence published in 2001 by the same authors increased the influence of PSO [32]. After that, a number of research papers and achievements related to PSO have appeared in the literature [33,34,35]. Basically, for the PSO algorithm, the search space of the optimisation problem is similar to the flying space of a flock of birds finding food. All the birds in the flock are seen as particles which represent possible solutions to the problem. The optimum solutions are similar to the food location of the bird flock. The movement of all the particles follows simple mathematical rules similar to bird flock movement. Therefore, an optimisation problem can be solved by iteratively improving the candidate solutions. All the particles are randomly initialised at the beginning. Then in each iteration, the value of each particle is updated according to its own best-known position i.e., personal best value and the best-known position in the particle swarm i.e. the global best value. The global best value is updated when better positions are found by other particles. The swarm will finally converge to a single optimum solution after a certain number of iterations. At each iteration, *t*, the positions and velocities of all particles are updated by Equations (1) and (2) which are termed update equations or flight equations [36]:

(1) $$v_{ij}^{t+1}=wv_{ij}^{t}+c_{1}r_{1}^{t}\left[p_{ij}^{t}-x_{ij}^{t}\right]+c_{2}r_{2}^{t}\left[p_{gi}^{t}-x_{ij}^{t}\right],$$

(2) $$x_{ij}^{t+1}=x_{ij}^{t}+v_{ij}^{t+1},$$

where *i* represents the *i^th^* particle of *N* particles in the swarm and *j* represents the *j^th^* dimension of the *D* dimensional search space. *t* is the current number of iterations. *v* and *x* are the velocity and position of the particle respectively. *p_ij_* is the personal best position of a particle. *p_gi_* is the global best position of the swarm. *r*_1_ and *r*_2_ are two random functions in the range from zero to one. It can be seen from Equations (1) and (2) that the new position of a particle is determined by the velocity of the particle, which is affected by three terms. The first term is the current velocity of the particle which is weighted by inertia weight, *w*, it represents the habit of movement of a particle and shows the trend for a particle to maintain its velocity in the previous iteration. The second term is the cognition part, which is weighted by the cognitive acceleration coefficient, *c*_1_. This term represents the memory of searches in previous iterations of a particle and indicates the trend for a particle to move towards its personal-best position. The third term weighted by the social acceleration coefficient, *c*_2_, is the social part, which represents the collaboration among the particles, and indicates the trend for a particle to move towards the global best position of the swarm. The inertial weight, *w*, can be used to control the global search capability. High values of *w* increase global search capability and low values increase local search capability. Usually, *c*_1_ is set equal to *c*_2_ to ensure the same influence of personal and global experience, so that a better solution can be obtained [23].

### 2.3. Overview of Particle Swarm Optimisation Variants

This paper employed three variants of PSO which have been compared in detail in a case study of a U.S. HEMS which was conducted by the authors. The three variants are CSPSO, QPSO and QPSOL. These PSO variants are introduced respectively in this subsection.

#### 2.3.1. Crossover Subswarm PSO (CSPSO)

CSPSO was proposed by the current authors in a U.S. case study by combining the improved crossover method and subswarms method. Crossover operations are inspired by a Genetic Algorithm (GA) to diversify the particle swarm so that the global search capability is increased and the probability for particles to be trapped in local minimal solutions is reduced. The crossover method is developed by Park et al. [37]. A crossover rate, CR, is preset and then a uniformly distributed random number, *r*, is generated between zero and one. In any iteration, for each particle in each hour of the day, if *r* ≤ CR, the particle value stays the same. Otherwise, the particle value is replaced by the personal best value of the corresponding hour of the day. When CR is one, no crossover is conducted, the algorithm becomes the standard PSO. When CR is zero, the crossover is always applied, which is similar to GA. This crossover method aims to improve the personal best value of each particle. Based on the work of Park et al. [37], an improved crossover method was proposed by the current authors in a U.S. case study to further improve the standard PSO. The proposed method performs crossover using Park et al. method [37] when the personal best values stay the same for a certain number of iterations.

The current work utilised the subswarms method proposed by Pedrasa, Spooner and MacGill [38]. This method employs a number of subswarms to perform optimisation in the search space. Each subswarm will have the same particle number as the original particle swarm and the subswarms work independently of each other. Each subswarm produces a global optimal solution. These best solutions are compared among all subswarms and the solution with the best fitness value of the objective function is chosen as the final solution of the algorithm. The CSPSO was developed by combining the improved crossover method and subswarms method.

#### 2.3.2. Quantum Particle Swarm Optimisation (QPSO)

The traditional QPSO proposed by Sun et al. is utilised in this paper [39]. In QPSO, the movement of particles follows quantum mechanics. The principle of QPSO is based on the fact that in standard PSO each particle, *i*, converges to its local attractor, *p*, given in Equation (3):

(3) $$p=\left(r_{1}p_{ij}+r_{2}p_{gi}\right)/\left(r_{1}+r_{2}\right),$$

where *p_ij_* and *p_gi_* are the personal best value of the particle and global best value of the swarm respectively. *r*_1_ and *r*_2_ are two random numbers that are uniformly distributed between zero and one. The local attractor, *p*, of the particle, *i*, stochastically exist in a hyper-rectangle with *p_ij_* and *p_gi_* being two ends of its diagonal. *Len* is the characteristic length of Delta potential well and can be calculated using Equation (4). Delta potential well is one of the paradigm potential field model in quantum mechanics [39]:

(4) $$Len=\frac{1}{g}\times \left|x_{ij}-p\right|,$$

where g is a parameter that is constrained by Equation (5):

(5) $$g>ln\sqrt{2},$$

Parameter *Len* can be controlled by altering parameter g. The formula to update the position of particles is shown in Equation (6):

(6) $$x_{ij}=\{\begin{array}{c} p-Len\times ln\frac{1}{\;r_{1}\;},\;\;if\;r_{2}>0.5\\ p+Len\times ln\frac{1}{\;r_{1}},\;\;\;\;\;otherwise \end{array},$$

where *r*_1_ and *r*_2_ are two random numbers that are uniformly distributed between zero and one. The algorithm of QPSO can be described as below:

(1) Randomly initialise the particle swarm.
(2) Determine the initial personal optimal and global optimal solution.
(3) For each dimension of each particle calculate *p* and *Len*.
(4) Update the position of each particle in the swarm.
(5) Update personal optimal and global optimal solution.
(6) Before the preset number of iterations is reached, repeat from step 3 to 5.

By combining with the improved crossover method and the subswarms method, the QPSO was further improved by the current authors in the U.S. case study.

#### 2.3.3. Quantum Particle Swarm Optimisation Procedure with Lévy Flights (QPSOL)

Grasso and Borean proposed the QPSOL on the basis of QPSO [40]. QPSOL assumes that the particles move around their attractor within a probability distribution similar to QPSO. However, instead of using the exponential distribution in quantum physics, the movement of particles is governed by the nature-inspired Lévy distribution. A number of studies have indicated that the typical characteristics of the Lévy probability distribution exist in the random walk behaviour of many animals and insects and the random walk is termed Lévy flight [40,41,42,43]. A random number with Lévy distribution can be generated using Equation (7) [41]:

(7) $$Lévy\;\left(\alpha \right)\sim \frac{\varphi \times d}{{\left|f\right|}^{1/\alpha }}$$

where *d* and *f* are drawn from the standard normal distribution. *α* is called the index of stability which determines the shape of the Lévy distribution with a value range of 0 < *α* ≤ 2. *φ* is calculated using Equation (8).

(8) $$\varphi ={\left(\frac{\Gamma \left(1+\alpha \right)\times sin\left(\pi \times \alpha /2\right)}{\Gamma \left(\left(\frac{1+\alpha }{2}\right)\times \alpha \times 2^{\left(\alpha -1\right)/2}\right)}\right)}^{1/\alpha },$$

In QPSOL, the Lévy flight is performed with Equation (9).

(9) $$\begin{array}{l} p=rp_{ij}+\left(1-r\right)p_{gi}\\ x_{i\;}=\;p+\beta \left(p-x_{i}\right)\lambda_{i} \end{array}$$

where *r* is a uniformly random number in the range from zero to one. *x_i_* is the particle position. *λ_i_* is a random number with Lévy distribution and *β* is the constriction coefficient that changes the step size of the flight.

By combining with the improved crossover method and the subswarms method, the QPSOL was further improved by the current authors in the U.S. case study.

### 2.4. Derivation of Indoor Thermal Model

**Figure 1:**
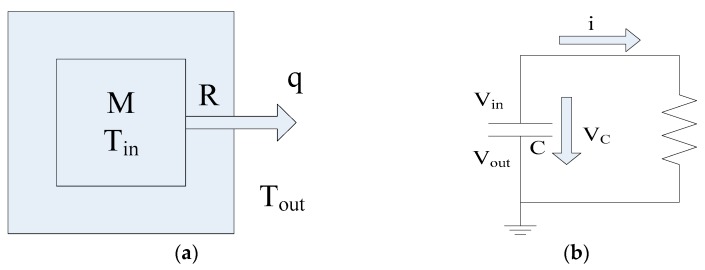
(**a**) Indoor thermal model; (**b**) Electrical equivalent model.

In order to update the indoor temperature every hour under the influence of the HP, an indoor thermal model has been established by developing an equivalent electrical model [44]. For this model, only heat conduction is considered neglecting heat convection and heat radiation. The outdoor temperature *T_out_* is set constant and the indoor temperature *T_in_* is uniform throughout the house. The indoor thermal model and its electrical equivalent model are given diagrammatically in Figure 1.

In the thermal model, the heat flows from the indoor environment with thermal mass, *M* (kWh/°C), and high temperature, *T_in_* (°C), to the low temperature outdoor environment with low temperature, *T_out_* (°C). The rate of heat flow is *q* (kW).

The electrical equivalent circuit consists of a charged capacitor, *C* (farad), discharging through a resistor, *R* (ohm). *V_in_* and *V_out_* are the voltages (volt) above and below the capacitor. The discharging current is *i* (amp). The elapsed time is *t* (s).

According to Kirchhoff’s law $V_{R}=V_{c}$, $V_{R}=iR$, $i_{c}=-C\frac{dV_{c}}{dt}$, therefore:

$$RC\frac{dV_{c}}{dt}\;+\;V_{c}\;=\;0$$

$$V_{c}=\;V_{0}e^{-t/\tau }$$

where $V_{0}=\;V_{in}-V_{out}$ and τ=RC.

As $V_{c}=\;V_{in}\left(t\right)-V_{out}$, where *V*_in_ is a function of time *t*:

$$V_{in}\left(t\right)=V_{0}e^{-t/\tau }+V_{out}$$

$$V_{in}\left(t\right)=\left(V_{in}-V_{out}\right)e^{-\frac{t}{RC}}+\;V_{out}$$

Therefore, the corresponding thermal equation is

$$T_{in}\left(t\right)=\left(T_{in}-T_{out}\right)e^{-\frac{At}{M}}+\;T_{out}$$

where *A* is the overall thermal conductivity in kW/°C. This equation can be rewritten as

$$T_{in}\left(t\right)=\varepsilon T_{in}+\left(1-\varepsilon \right)T_{out}$$

where $\varepsilon =e^{-t/\tau }$, and *ε* is called the factor of system inertia.

When a cooling or heating energy source, *e* (kW), is present, and the efficiency (COP of a HP) is *η*. The actual cooling or heating power is *ηe* (kW), and the above equation becomes Equation (10).

(10) $$T_{in}\left(t\right)=\varepsilon T_{in}+\left(1-\varepsilon \right)(T_{out}\mp \frac{\eta e}{A})$$

where $+\frac{\eta e}{A}$ is used for a heating energy source and $-\frac{\eta e}{A}$ is used for a cooling energy source.

The derived Equation (10) is the same as the space conditioning model developed by Constantopoulos et al [45], which therefore proves the correctness of the derivation process.

### 2.5. Measured Heat Pump Performance Data

This current study utilises HP related data from the UK Government’s Renewable Heat Premium Payment (RHPP) scheme, which ran from 2011 to 2014. This data set is available on the UK data archive [46]. This data set is validated and filtered by Research and Analysis on Performance and Installation Data–Heat Pump Consortium (RAPID-HPC). The potential anomalies in the data set were filtered. This data set contains electricity consumption and heat output of HPs for two-minute intervals covering 696 different sites. These sites were located throughout the UK. The metering start date is different for each site within the overall trial period. Each site has around two years of data. 328 properties were registered social housing and the remainder were owner-occupied properties. A range of dwelling construction types, sizes, ages and households are covered in this data set. The monitored data are adjusted to a standard set of weather conditions in order to compare different weather regions across the UK and remove the influence of the warmer than average winter that occurred during the period of trial. As in the Standard Assessment Procedure (SAP) the East Pennines is the area used for weather data, the monitored data is adjusted to the 10 year (1998–2007) mean temperature observed in the East Pennines. As the exact location used to derive the SAP data is unclear, the weather data from Grantham, Lincolnshire over the 10 year period has been chosen as the monthly mean values of weather data closely resemble those specified in SAP. As outdoor weather conditions were not monitored in the RHPP scheme, data from the United States National Centres for Environmental Prediction (NCEP) Climate Forecast System Reanalysis is utilised [47]. This weather data set is accurate and compares well with other historic weather data sets. Furthermore, it provides a higher geographical resolution with no missing data as recommended by the HP data set description document.

**Figure 2:**
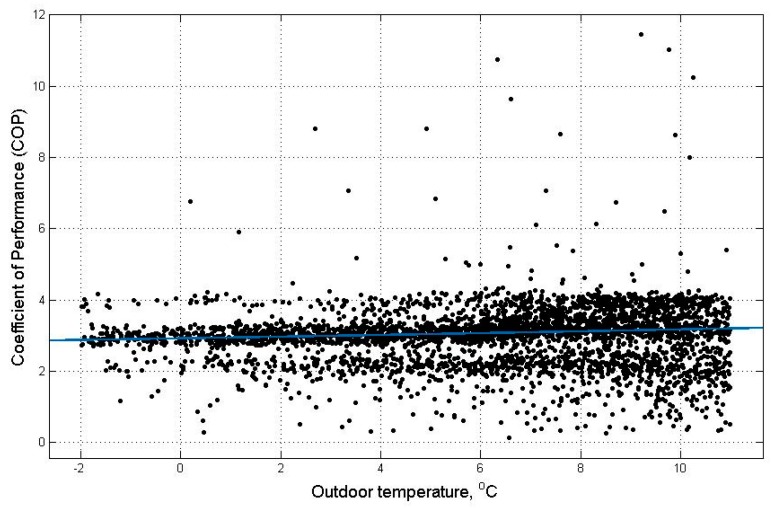
Coefficient of Performance (COP) versus outdoor temperature for a heat pump operating over a one-year period.

**Figure 3:**
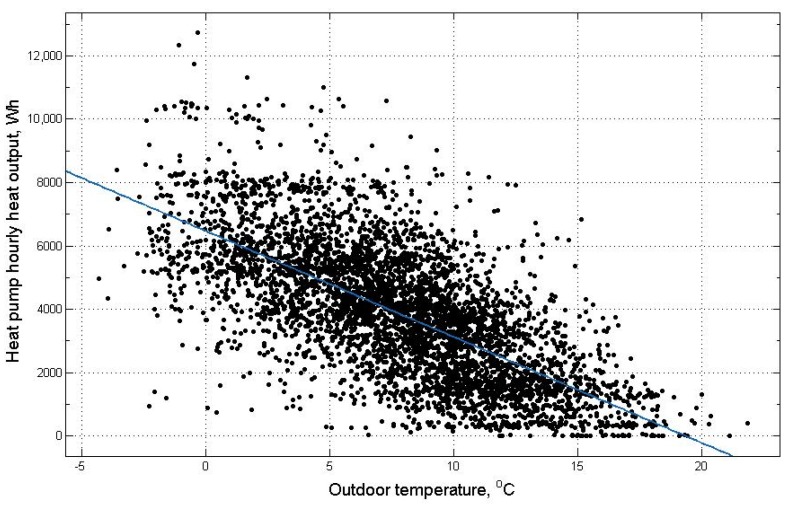
Heat pump hourly heat output versus outdoor temperature for a heat pump operating over a one-year period.

This current study selected a domestic ground source HP in a detached house. The property was built between 1945 and 1964 with more than four bedrooms. About one year’s data of electricity consumption and heat output of the HP is utilised together with the corresponding outdoor temperature data at Grantham, Lincolnshire to generate characteristic curves of COP versus outdoor temperature and heat pump hourly heat output versus outdoor temperature. The chosen one year’s data starts at 1-2-2014 and ends at 14-1-2015. In order to match the hourly outdoor temperature data [47], the two-minute interval data of electricity consumption and heat output of the HP were aggregated to hourly time intervals. For each hour, the mean of the two-minute intervals was calculated and multiplied by 30 to reach an hourly average. The characteristic curves were generated using the curve fitting tool box in MATLAB. The COP versus outdoor temperature curve is shown in Figure 2 and the heat pump hourly heat output versus outdoor temperature curve is shown in Figure 3. The black data points in Figure 2 and Figure 3 are the original measured data over the one-year period. The data was conditioned with unreasonable error data removed and the data when the HP heat output is zero or very low values that are close to zero were removed leaving data when the HP is actually operating. For the COP versus outdoor temperature curve, the number of data points available for higher temperature is a lot less than the number of data points for lower temperature due to the intrinsic feature of the UK climate. As the number of data points is small for higher temperatures, the resulting trend curve is not fully capturing the performance of the HP at higher temperatures. In addition, when the outdoor temperature is high, the HP produces little or no heat as there is no demand but the HP consumes electricity to remain in the on state. This can lead to low COP values. In order to eliminate the influence of imbalance of data density between high and low temperatures, the COP data are deleted when the outdoor temperatures are above 11 °C. The data points for the HP COP are widely spread out for the two curves. For the COP versus outdoor temperature curve, the COP values are not only affected by the outdoor temperature but also affected by the Part Load Ratio (PLR) [48]. The PLR can be calculated as the ratio of the actual heat output of the HP to the maximum heat output that could be generated by the HP in a certain time interval [49]. The heat demand varies for different times of the year and the PLR varies accordingly causing the COP to vary. For the heat pump hourly heat output versus outdoor temperature curve, the data points are spread out as there are some temperature independent demands at different times [50]. For example, cooking activities and boiling water. Heat demand can also be affected by the number of people in the house. The data points in the two curves can also be subjected to the influence of various other real-world factors.

The equation obtained for the COP versus outdoor temperature curve is shown in Equation (11):

(11) $$f\left(x\right)=p1\times x^{2}+p2\times x+p3$$

where f(x) represents COP and x represents outdoor temperature. The coefficient values are: *p*1 = 0.0002377, *p*2 = 0.02272, *p*3 = 2.922.

The goodness of fit is represented by R-square of 0.9616, Adjusted R-square of 0.9616. R-square can be any value between 0 and 1, with a value closer to 1 indicating that a greater proportion of variance of the data is represented by the fit. The adjusted R-square can be any value less than or equal to 1, with a value closer to 1 indicating a better representation of the data. Least absolute residuals (LAR) is selected as a robust option for curve fitting. The absolute difference of the residuals of the generated curve is minimised by the LAR method, rather than the squared differences. Thus, outliers have a lesser influence on the fit. The polynomial curve of degree 1 and 2 were compared and the degree 2 curve was chosen as higher R-square and adjusted R-square value can be achieved.

The equation obtained for the heat pump hourly heat output versus outdoor temperature curve can be represented by Equation (12) which is a simple straight line equation:

(12) f(x)=p1×x+p2

where f(x) represents heat pump hourly heat output and x represents outdoor temperature. The values for the coefficients are: *p*1 = −334.7, *p*2 = 6471.

The goodness of fit is represented by R-square of 0.4867, Adjusted R-square of 0.4866. The bisquare weight method is selected as a robust option for curve fitting. This approach minimises a weighted sum of squares. The weight given to each data point is determined by the distance from the point to the fitted line. Full weight is given to the points near the line and reduced weight is given to points far from the line. Points that are very far from the line could be given zero weight. This approach tries to find a curve that fits the majority part of the data while minimising the influence of outliers.

Five cold winter days in 2019 were chosen for the simulation using the HP control program. The dates were chosen as they are among the coldest days in the winter when the HP was frequently used. The details of the dates are in Section 3. The COP and heat pump hourly heat output data for the chosen dates were generated using the two equations of the characteristic curves based on the outdoor temperature data for the days [47]. The hourly electricity consumption of the HP is calculated using the generated COP and heat pump hourly heat output data. The TOU electricity day ahead auction prices for the UK for the chosen dates are used in the HP control program [51].

### 2.6. Program Description

The HP control program is developed using C# in the Microsoft Visual Studio 2017 environment. The program serves as part of the HEMS. The full HEMS optimisation program will incorporate other typical loads, renewable energy (solar and wind) and a battery system. The aim of the developed program is to determine the optimal temperature setting for the HP to operate on an hourly basis for 24 h and minimise the electricity cost while maintaining the users’ comfort at the same time. The outdoor temperatures, real-time electricity price data on the chosen dates are used as mentioned in the data section. The electric power rating of the HP is not given in the HP data set. The maximum hourly electricity consumption in the one year measured HP data is 8.157 kWh. However, the hourly electricity consumption is the average electricity consumption of the hour and instant electricity consumption could be higher. Therefore, the power rating of the HP is assumed to be 9 kW.

As concluded by AECOM (a multinational Architecture, Engineering, Consulting, Operations and Maintenance firm) and the UK department for communities and local government, the literature about how UK dwellings modify outdoor temperatures is dominated by modelling studies and the available published measured data is rare [52]. Renaldi, Kiprakis and Friedrich also indicated that a critical input to a heating system optimisation framework is the heat demand data. However, a real measured demand profile with complete supporting information is difficult to obtain and scarce in the literature. As a result, Renaldi, Kiprakis and Friedrich employed a heat demand model to generate heat demand profiles [53]. Navarro-Espinosa et al. also generated profiles for loads and electric heat pumps using a domestic energy consumption model [54].

Unfortunately, the heat pump data from the RHPP scheme utilised in this current work and the supporting indoor temperature information is not readily available. As there is no measured indoor temperature data for the chosen HP on the chosen dates, the indoor temperatures are generated using the temperature update model i.e., Equation (10). In the temperature update model, the factor of system inertia, *ε*, is taken as 0.93 [55], the overall thermal conductivity, *A*, is calculated as 0.27 kW/°C. In order to determine parameter *A*, a fabric and ventilation heat loss calculation is conducted for a typical UK house of 226 m^2^. The typical U values for the walls, ceiling and windows are used to conduct calculations to UK standards. The result of overall thermal conductivity is 0.27 kW/°C. To validate the correctness of this value, simulations were conducted on the temperature update model i.e., Equation (10) using outdoor temperatures and the heat pump hourly heat output data for the chosen HP for a month between 3-1-2019 to 3-2-2019. A temperature of 18 °C is recommended by the 2016 Cold Weather Plan for England as day and night minimum temperature for those 65 and older or anyone with pre-existing medical conditions [56]. For houses insulated to typical UK levels, the on/off set-point for the heating system is usually between 19 to 23 °C [57]. Thus, the initial temperature at midnight is assumed to be 19 °C. When *A* is 0.27 kW/°C, among all indoor temperature data generated, most data are within the typical range of 19 to 23 °C [57]. Additionally, 2% of the data are above 23 °C but below 23.5 °C. No data are below 19 °C. Thus, all data generated are above the 18 °C which has been defined as the minimum temperature [56]. Therefore, this *A* value is considered suitable for a UK housing condition. The indoor temperatures on the chosen dates were then generated using the temperature update model assuming the initial temperature at midnight is 19 °C.

The optimal comfort range of indoor temperature for the HP control program is set between 19 and 23 °C in order to compare with the unscheduled case of the data as this is the typical range of set-point for heating systems in the UK and the indoor temperature range in the data [57]. The finish condition of the program is that the number of iterations reaches 2000 and the temperature is perfectly kept within the set range. PSO is used as the optimisation algorithm for the program. During each iteration, each particle is evaluated using the final objective function, *F_hp_*. The final objective function is constructed as shown in Equation (13):

(13) $$F_{hp}=Obj+Pena$$

where *Obj* is the objective function and *Pena* is the penalty function. The objective function *Obj* is constructed as shown in Equation (14).

(14) Obj=a×Pri+(1−a)×Comf

where *Pri* is the total electricity cost of the HP for 24 h. *Comf* is the comfort factor of the end users which is calculated according to the level of deviation of the ideal temperature set by the end users. A *Comf* value of 0 indicates that the temperature is perfectly within the set temperature range and a larger *Comf* value indicates the larger deviation. The preference factor, *a*, is set by the end users to trade-off between comfort and cost. The penalty function is constructed to penalise when the hourly electricity consumption is more than the maximum electricity consumption allowed by the HP, which is 9 kW for this study.

The aim of the program is to minimise the final objective function. In other words, the aim is to minimise the total electricity cost, *Pri*, and the comfort factor, *Comf*. Finally, the program outputs the optimal temperature setting of the HP of the 24-hour-day, which minimises the electricity cost of the end users while maintaining their comfort level.

**Figure 4:**
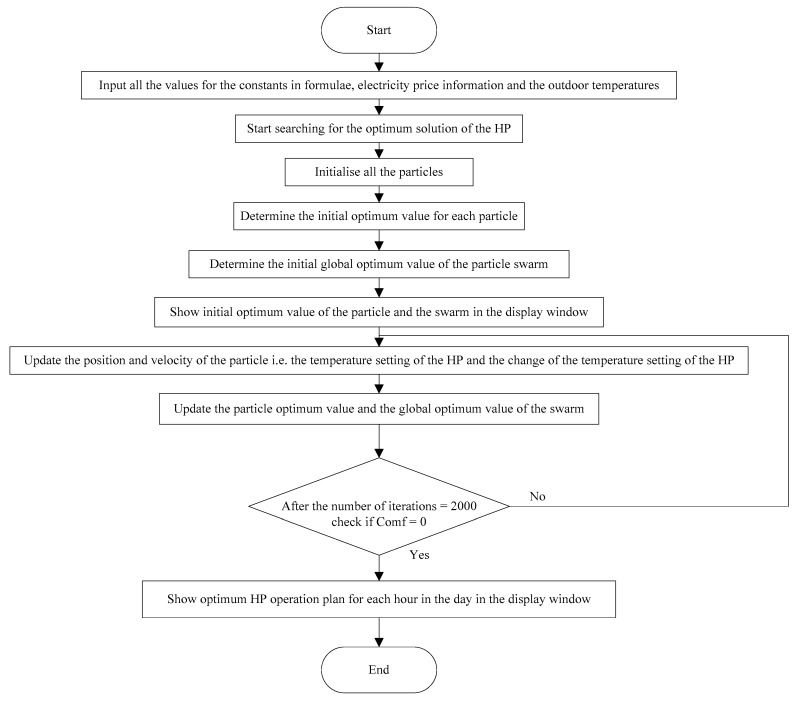
Flowchart of HP control program using PSO.

The flowchart of the program is shown in Figure 4.

## 3. Results and Discussion

The test results for PSO and its variants are discussed and compared with the unscheduled result from the data in this section. PSO and its variants are all stochastic search algorithms. In order to reduce the stochastic elements of the results, the average value of the results for 50 searches is taken as the results in this paper.

### 3.1. The Results of the Standard Particle Swarm Optimisation 

**Figure 5:**
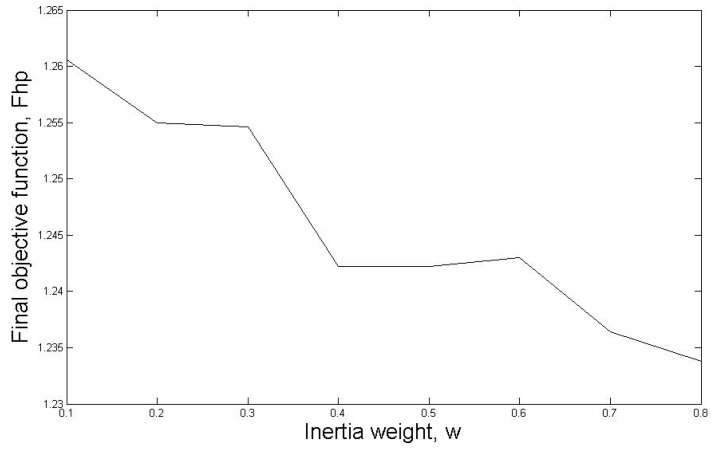
The relationship between inertia weight, *w*, and the final objective function, *F_hp_*.

In standard PSO, the effect of varying the value of the inertia weight, *w*, is investigated for five cold winter days in 2019. The details of the results for 3-2-2019 is shown. The relationship between inertia weight, *w*, and the final objective function, *F_hp_*, is shown in Figure 5.

It can be seen that final objective function, *F_hp_*, gradually decreases from 1.261 when inertia weight *w* = 0.1 to the lowest value of 1.233 when inertia weight *w* = 0.8. When inertia weight *w* = 0.8, the total electricity cost after optimisation using standard PSO is compared with the actual electricity cost in the data. For the chosen data, the total electricity consumption for the HP during the 24 h on 3-2-2019 is €3.13. When inertia weight *w* = 0.8, the total electricity cost is €2.47. The saving is €0.66, which is 21.09% cost reduction compared with the actual cost in the data. The average search time of standard PSO is 1089 ms.

The same parameter setting i.e., *w* = 0.8 were applied for optimisation of four other cold winter days in 2019. The results of the five days are summarised in Table 1.

It can be seen that the standard PSO has a stable performance for different cold winter days. The average percentage of cost reduction compared with the electricity cost in data for the five days is 21.09%.

### 3.2. The Results of the Crossover Subswarm PSO

The CSPSO algorithm proposed by the authors in another U.S. case study is utilised. The details of the results for 3-2-2019 is as follows. The best result achieved by this algorithm is 1.14 of the final objective function, *F_hp_*, which occurred when the number of subswarms is 50 and crossover rate, CR, is 0.3. Crossover is applied when personal best values haven’t changed for 10 iterations. 50 particles are used in each subswarm. The total electricity cost for the day is €2.28 which is €0.85 or 27.16% saving compared with the €3.13 cost in the data. The average search time of CSPSO for each subswarm is 1576 ms.

The same parameter setting were applied for optimisation of four other cold winter days in 2019. The results of the five days are summarised in Table 2.

The results show that the CSPSO algorithm has better optimisation performance compared to the standard PSO for different cold winter days. The average percentage of cost reduction compared with the electricity cost in data for the five days is 25.61%.

### 3.3. The Results of the QPSO and QPSOL

The details of the results of improved QPSO for 3-2-2019 is shown below. The best result for improved QPSO is 1.12 for the final objective function, *F_hp_*, when the total electricity cost is €2.25 and parameter g is set to 0.9. The number of particles in each subswarm is 30. The number of subswarms is 50. Although the optimisation result of improved QPSO is slightly better than CSPSO, the search time of improved QPSO for each subswarm is 6400 ms, which is about four times CSPSO.

The same parameter settings were applied for optimisation of four other cold winter days in 2019. The results of the five days are summarised in Table 3.

It can be seen that the improved QPSO algorithm achieved slightly better optimisation results compared to the CSPSO for different cold winter days. The average percentage of cost reduction compared with the electricity cost in data for the five days is 26.76%.

The details of the results of improved QPSOL for 3-2-2019 is as follows. The best result for improved QPSOL is 1.12 for the final objective function, *F_hp_*, when the total electricity cost is €2.25. Index of stability, *α*, is 1.4 and constriction coefficient, *β*, is 0.6. The number of particles in each subswarm is 30. The number of subswarms is 60. Although the optimisation result of improved QPSOL is slightly better than CSPSO, the search time of improved QPSOL for each subswarm is 18252 ms, which is about 11 times CSPSO.

The same parameter settings were applied for optimisation of four other cold winter days in 2019. The results of the five days are summarised in Table 4.

The results indicate that the improved QPSOL algorithm is able to achieve similar optimisation results compared to the improved QPSO algorithm for different cold winter days. The average percentage of cost reduction compared with the electricity cost in the data for the five days is 26.93%.

## 4. Discussion 

The results of the standard PSO and its variants after improvement are compared in Table 5.

It can be seen that the CSPSO, improved QPSO and improved QPSOL all achieved better results compared with the standard PSO in terms of the average percentage of cost reduction compared to the cost in the data for five different cold winter days in 2019. However, the search times of improved QPSO and improved QPSOL are much longer than CSPSO due to the complex calculation of quantum mechanics.

Among the works in the literature, Gudi et al. increased the efficiency of energy utilisation by 16.4% by using the proposed PSO approach for a dynamic DER management system [7]. Gudi, Wang and Devabhaktuni proposed an optimised resource management strategy using PSO and achieved cost reduction by 16–17% [14]. Zhao et al. achieved daily electricity cost reduction for the overnight heating, daytime heating, and evening heating for around £0.33, respectively, compared with the unscheduled case without HEMS, which is 4.86%, 4.43%, and 4.15% reduction, respectively. The mixed integer linear programming was utilised for optimisation [19]. All these papers in the literature have different system structure and settings. The aims of optimisation for these works are not exactly the same but are all within the HEMS optimisation context. The results of these papers cannot be directly compared. However, in terms of percentage of electricity cost reduction compared to the unscheduled case before optimisation, the current research utilised the CSPSO algorithm proposed by the authors in another U.S. case study and achieved an average percentage of electricity cost reduction of 25.61% with an acceptable length of search time for five cold winter days in 2019. The achieved average percentage of electricity cost reduction is better than the work mentioned in literature. Furthermore, none of the research in the literature has adopted particle swarm optimisation algorithm to optimise HP operations in a HEMS. The PSO algorithm consumes less computational resources compared with the mathematical optimisation methods like mixed integer linear programming and mixed integer nonlinear programming [15,16,19,22]. Comparing with complicated Multi-agent based architecture using MATLAB [17], the PSO approach is simple to program with several lines of code. The PSO approach makes full use of the hourly changing TOU electricity price to adjust the temperature set-point of the HP within a preset comfort range (19 to 23 °C) rather than simple set-point temperature control which can only change the thermostat set-point by 1 degree Celsius according to a preset electricity price threshold [20].

## 5. Conclusions

This paper presents a purposely-developed HP control program using the PSO algorithm and its variants and derived the indoor thermal model for temperature update in the program. There is no details in the literature of any authors who have adopted particle swarm optimisation algorithm to optimise HP operations in a HEMS. The optimisation algorithms utilised in the literature were either complicated and require heavy computation or too simple and not sufficient for the optimisation problem. Therefore, this paper adopted the PSO based methods which are simple and efficient. The operational cost of the HP is minimised while maintaining end-user comfort levels. This is achieved by optimising the temperature setting of the HP. Characteristic curves of COP versus outdoor temperature and heat pump hourly heat output versus outdoor temperature are generated and the corresponding equations are obtained based on one year’s HP measured data. These characteristic curves were then utilised to generate COP and heat pump hourly heat output data for any time when the outdoor temperature data is available. Additionally, the hourly electricity consumption of the HP can be calculated using the generated data. This paper adjusted the parameters of the indoor thermal model to adapt it to the UK house condition using a month of data in winter 2019. The adjusted model outputs data that are mostly within the typical indoor temperature range in the UK with 2% of the data above but within 0.5°C of the upper limit of the typical indoor temperature range of 19 to 23 °C. This paper compared different PSO variants with standard PSO and the unscheduled case calculated from the data. Comparing with the unscheduled electricity cost calculated using the data, the standard PSO achieved 21.09% cost reduction. The CSPSO, improved QPSO and improved QPSOL achieved 25.61%, 26.76%, 26.93% cost reduction respectively, which shows improvement compared to the standard PSO. However, the search time of improved QPSO is about four times CSPSO, the search time of improved QPSOL is about 11 times CSPSO. Among all chosen algorithms, the CSPSO achieved good cost reduction of 25.61% with a short search time of 1576 ms for each subswarm. This result is better compared with a number of papers in the literature. The results of this work indicate that there is a huge potential to reduce the cost during HP operation while maintaining end user comfort levels. For future development, other typical loads will be included in the system. A solar PV system, an energy storage system and grid integration will also be considered in the system. The variations of parameter *A* that represent different building types in the UK will be investigated further.

## Figures and Tables

**Table 1:** Summary of results of the standard PSO for five cold winter days in 2019.

Data	3rd Feb.	31st Jan.	23rd Jan.	20th Jan.	18th Jan.	Average
Optimised electricity cost (€)	2.47	3.01	3.56	3.01	2.79	
Electricity cost in data (€)	3.13	3.81	4.53	3.82	3.52	
Percentage of cost reduction (%)	21.09	21.00	21.41	21.20	20.74	21.09

**Table 2:** Summary of results of the CSPSO for five cold winter days in 2019.

Data	3rd Feb.	31st Jan.	23rd Jan.	20th Jan.	18th Jan.	Average
Optimised electricity cost (€)	2.28	2.85	3.37	2.85	2.65	
Electricity cost in data (€)	3.13	3.81	4.53	3.82	3.52	
Percentage of cost reduction (%)	27.16	25.20	25.61	25.39	24.72	25.61

**Table 3:** Summary of results of the improved QPSO for five cold winter days in 2019.

Data	3rd Feb.	31st Jan.	23rd Jan.	20th Jan.	18th Jan.	Average
Optimised electricity cost (€)	2.25	2.81	3.31	2.77	2.64	
Electricity cost in data (€)	3.13	3.81	4.53	3.82	3.52	
Percentage of cost reduction (%)	28.12	26.25	26.93	27.49	25.00	26.76

**Table 4:** Summary of results of the improved QPSOL for five cold winter days in 2019.

Data	3rd Feb.	31st Jan.	23rd Jan.	20th Jan.	18th Jan.	Average
Optimised electricity cost (€)	2.25	2.8	3.32	2.77	2.61	
Electricity cost in data (€)	3.13	3.81	4.53	3.82	3.52	
Percentage of cost reduction (%)	28.12	26.51	26.71	27.49	25.85	26.93

**Table 5:** Results of the standard PSO and its variants after improvement.

	Standard PSO	CSPSO	Improved QPSO	Improved QPSOL
Average percentage of cost reduction (%)	21.09	25.61	26.76	26.93
Search time (ms)	1089	1576	6400	18252
